# Education researchers’ beliefs and barriers towards data sharing

**DOI:** 10.1007/s11135-025-02188-6

**Published:** 2025-04-29

**Authors:** Jessica A. R. Logan, Allyson L. Hayward, Lexi E. Swanz, Ayse Busra Ceviren

**Affiliations:** 1 Vanderbilt University, 110 Magnolia Circle 302B, Nashville, TN 37212 USA; 2 The Ohio State University, Columbus, Ohio USA

**Keywords:** Data sharing, Open science, Data sharing beliefs, Data sharing attitudes

## Abstract

Data sharing is increasingly becoming a highly encouraged or required practice for any federally funded research projects. However, the uptake of these practices in education science has been minimal. Research suggests that many researchers believe data sharing should be practiced always or often, but also suggests that many researchers rarely practice data sharing. This disconnect indicates a general lack of understanding around data sharing and suggests there are salient barriers that prevent education researchers from engaging in the practice. This work examines (a) the prevalence of positive attitudes and perceived barriers to data sharing in a sample of education researchers, and (b) if there is a difference between the perceived barriers for researchers who have different levels of data sharing experience. Results suggest education researchers generally hold positive attitudes towards data sharing, with 70% of the sample agreeing that it benefits their career, increases citations, and is good for science. However, barriers such as concerns about IRB issues and the potential for misinterpretation of shared data were prevalent among respondents. Additionally, researchers with more experience sharing data were less likely to agree with these barriers compared to those with less or no sharing experience.

**Supplementary Information:**

The online version contains supplementary material available at 10.1007/s11135-025-02188-6.

## Education researchers’ beliefs and barriers to data sharing

In recent years, the education sciences have witnessed growing momentum toward the public sharing of research data. The U.S. federal government has long championed open access to federally funded research, beginning with a 2013 memorandum advocating for increased access to scientific results (Holdren 2016). This commitment was reinforced and expanded in 2022 when the White House Office of Science and Technology Policy (OSTP) released an updated directive, emphasizing the need for immediate public access to research outputs. The 2022 policy underscored the principle that “there should be no delay between taxpayers and the returns on their investments in research,” while also shortening timelines for compliance.

Building on this federal mandate, funding agencies have evolved their policies to reflect the shifting landscape. The National Institutes of Health (NIH) introduced a new data management and sharing policy effective January 2023, and the Institute of Education Sciences (IES) announced similar updates for its 2025 funding cycle (Institute of Education Sciences 2024). Both policies require researchers to share data generated through federally funded projects and outline potential consequences, such as limitations on future funding, for non-compliance. These developments signal a clear direction for the scientific community: open data is no longer optional but an integral part of the research process. By prioritizing transparency and accessibility, these policies aim to maximize the impact of research investments and underscore the importance of making data widely available for broader scientific and societal benefit.

## Previous research on data sharing

Though the push for data sharing is relatively new to the field, education researchers seem to be generally supportive of the practice. In a survey of more than 1,500 education researchers, about 75% of respondents indicated that data sharing should be used almost always or often (Makel et al. 2021). In a similar survey of researchers in the field of special education, Fleming et al. 2022 found that only 28% of participants opposed data sharing. Despite this apparent support, education researchers frequently report low engagement in data sharing themselves. For instance, Makel et al. (2021) found that around 80% of education researchers had never or only occasionally shared their data. Likewise, Fleming et al. (2022) reported that only 11% of special education researchers had shared data in the past, although many expressed intentions to do so in the future. This discrepancy between general acceptance of data sharing and actual participation suggests that education researchers may face significant barriers to adopting this practice.

Previous studies in education and other fields have explored such barriers. One frequently cited barrier is a lack of knowledge. A 2022 study of 155 researchers in the field of Special Education found that 71% of respondents rated themselves as having low or no knowledge of data sharing (Fleming et al. 2022). Similar findings have been reported in other related social science fields. For example, Houtkoop et al. (2018) conducted a survey of 600 researchers in psychology and found that 54% indicated that they never learned to share their data online, and 63% indicated they would be more likely to share their data if someone would show them how.

Other barriers include time constraints and funding limitations, which have been reported across disciplines (e.g., Houtkoop et al. 2018; Tenopir et al. 2011). Concerns about the data depositor not being credited for their work are also present (Schmidt et al. 2016). Additionally, psychological researchers surveyed by Houtkoop et al. (2018) identified fears of misinterpretation (49%) and other risks, such as being “scooped,” intellectual property issues, unintended secondary use of data, and potential discovery of errors, with approximately 40% of respondents endorsing these concerns. Despite these reservations, many respondents expressed a willingness to share the data from their next publication in an online repository, suggesting that these barriers may not be insurmountable.

In summary, although the concept of data sharing has general support among researchers, actual participation remains low (Makel et al. 2021; Houtkoop et al. 2018; Fleming et al. 2022). This gap may reflect the influence of perceived barriers, such as insufficient knowledge, time constraints, or fears about data misuse. While previous work provides a framework to understand data sharing challenges, it is not clear whether or to what extent the same barriers would apply to researchers working in the field of education.

In this study, we sought to examine education researchers’ general agreement towards data sharing, the barriers they perceive related to data sharing, and whether perceived barriers to data sharing are associated with researchers’ data sharing experiences. Specifically, we ask:

1. To what extent do education researchers agree with positive statements about data sharing?
2. To what extent do education researchers agree with perceived barriers to data sharing?
3. Is there a difference between the perceived barriers for researchers who have different levels of data sharing experience?

## Methods

### Participants and recruitment

This work is a secondary analysis of a larger study that surveyed researchers on a wide range of topics related to data stewardship (see the preregistration for the larger study on the Open Science Framework (https://osf.io/8r746)). This secondary analysis was not itself preregistered, however we include the link to the parent study preregistration to demonstrate that key decisions around sample selection, data collection procedures, and inclusion/exclusion criteria were determined prior to any analysis.

We report how we determined our sample size, all data exclusions, and all manipulations done to the data (Simmons et al. 2012). We also report all analyses conducted in support of the ideas represented in this research paper. This includes one analysis that anonymous reviewers identified as not being well motivated, which is now presented in Supplementary Material 1.

For the larger study, anonymous survey links were distributed to potential participants after Institutional Review Board approval was granted. Survey invitations were sent through several platforms. First, to Special Education Research Accelerator (SERA) partners. SERA is a platform that organizes the collaboration of research teams to conduct crowd sourced replication studies. Members of SERA are affiliates of higher education institutions and have experience in data collection with students with or at risk for disabilities. It is a nationwide organization consisting of members from all nine U.S. census divisions. Second, invitations were sent to the listservs of three divisions of the American Education Research Association (AERA). While all division heads were solicited, Divisions A, B, and D agreed to include information about the project in their announcements. Third, the research team also used the award databases of IES and NSF to identify previously funded projects by researchers in the field of education; with invitations sent to primary investigators who received funding prior to 2020. A total of 1,114 emails were sent, with further invitations forwarded via listservs, though the exact number of forwarded invitations is unknown. Additionally, a link to the survey was posted on X (formerly known as Twitter). Respondents were asked to complete the anonymous survey only if they identified themselves as (1) an educational researcher and (2) a primary investigator or co-investigator on at least one published quantitative research article. The survey took approximately 15 min to complete. All participants who completed the survey were invited to enter in a drawing to win one of two $100 Amazon gift cards. Contact information for the prize drawing was collected on a separate form than the survey to uphold anonymity. Preregistration of the primary study indicated the survey would remain open and accessible for three months, or until it had accumulated over 200 responses, which ever occurred second. At the end of the three months (September–November 2021), we received 252 responses. Per exclusionary rules in the primary study’s preregistration, we excluded 74 respondents who filled out less than half of the survey or did not answer the items of interest to this study. This step was taken to ensure data quality and reliability and to prevent the introduction of bias and inaccuracies in the findings, resulting in a final sample of 178 respondents. Data used in the present study are shared at (https://ldbase.org/projects/dbf4158d-5e7b-407e-993d-da559c189a93). Code used to analyze the data is shared in Supplementary Material 2.

### Materials

The larger survey was developed by part of the author team. The items were developed by the researchers based on a thorough review of relevant literature and widely accepted views on data sharing, and reflect common perspectives on the topic. The entire survey was reviewed by an education researcher with extensive experience in the field, and feedback was incorporated to ensure clarity and precision in the items presented.

While the larger survey included questions on multiple factors, in this paper we focus on items related to data sharing. Specific wording for each item is listed in Table 1. Participants were asked to what degree they agree or disagree with each item using a 6-point Likert scale (strongly disagree, somewhat disagree, slightly disagree, slightly agree, somewhat agree, strongly agree). When examining descriptive statistics, we define “agreement” as a response of 4, 5, or 6 on the Likert scale.

**Table 1:** Mean agreement of survey items by group

Question	Overall M(SD)
Data sharing is good for my career	4.16 (1.33)
Data sharing will increase citations of my work	4.01 (1.29)
Data sharing is good for science	5.33 (0.89)
My IRB will have a problem with me sharing data	3.78 (1.58)
Data sharing is time consuming and expensive	3.55 (1.38)
If someone reuses my data, they may misinterpret it	3.81 (1.34)
I don’t know where to share my data	3.37 (1.75)
If I share my data, it might be possible to identify a participant	2.86 (1.84)
If I share my data, someone might publish my key findings before me	3.10 (1.66)
I don’t know how to start sharing my data	3.27 (1.71)
I don’t want to share my data because someone might find a mistake	2.65 (1.53)
These data exist because of my hard work, why would I share so someone else benefits	2.30 (1.42)

Note: Questions were answered on a Likert scale from strongly disagree (1) to strongly agree (6)

## Results

### Descriptive results

Demographic information about the sample is included in Table 2. Most respondents (76%, *n* = 135) identified their field of study as education, with 12% selecting psychology and another 12% selecting “other” (e.g., speech-language pathology, learning sciences, and hard sciences). The sample primarily consisted of early- to mid-career researchers (mean = 8.7 years of experience; median = 6 years; range = 1–30 years), with 86% reporting employment in academia or at a university. Regarding funding experience, 31% of participants had received funding from the Institute of Education Sciences (IES), 19% from the National Institutes of Health (NIH), and 40% from the National Science Foundation (NSF).

**Table 2:** Demographic information by sharing belief and experience

Variable	Overall		Sharing Experience						
			Collaborator (*n* = 72)		Repository (*n* = 74)		Neither (*n* = 31)		
	*n*	%	*n*	%	*n*	%	*n*	%	
Field (Select all that apply)									
Education	135	76%	55	41%	53	40%	26	19%	
Psychology	21	12%	8	38%	12	57%	1	5%	
Other	22	13%	9	43%	9	43%	3	14%	
Type of Workplace									
Academia	153	86%	64	42%	61	40%	27	18%	
Industry	6	3%	1	17%	5	83%	0	0%	
Research Institute	8	4%	2	25%	5	63%	1	13%	
Other	11	6%	5	45%	3	27%	3	27%	
Funded as a PI or Co-PI through IES, NIH, and/or NSF									
0	73	41%	25	35%	31	43%	16	22%	
1	33	19%	16	48%	12	36%	5	15%	
2+	72	40%	31	43%	31	43%	10	14%	
Planning to share data at the end of a current project									
Yes	76	43%	21	28%	47	62%	8	11%	
No	45	25%	25	56%	8	18%	12	27%	
I don’t know	57	32%	26	46%	19	34%	11	20%	

Note: The Overall column shows percentages within category. The Collaborator, Repository, and Neither column percentages represent the percentage of respondents within the row

Participants were also asked about their prior data sharing experiences. Two survey questions assessed this: (1) “How many times have you shared data from projects with a new collaborator?” and (2) “How many times have you posted data in a data repository?” Responses were rated on a scale of 1 (never) to 5 (75–100% of projects). To simplify analysis, responses for each question were collapsed into two categories: never shared (1) or had shared (2–5). We next combined the two questions for analysis, 40.6% of respondents had only shared data with collaborators, 41.8% had shared in a repository, and 17.5% had never shared data.

Despite most of the sample having shared before, intent for future data sharing was more variable. When asked if they were planning on sharing data from any current study once completed, 43% said yes, 25% said no, and 32% said they didn’t know.

### Research question 1: agreement with positive beliefs about data sharing

**Fig. 1:**
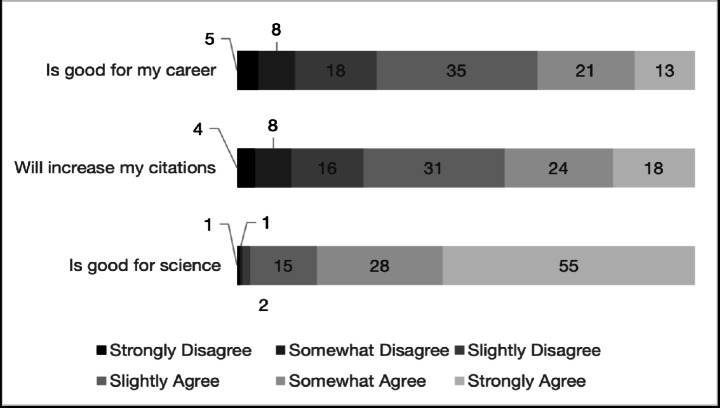
Percentage of the sample indicating each level of agreement with beliefs about data sharing

Participants generally expressed positive beliefs about data sharing. Table 1 shows the statements and their mean agreement levels, and Fig. 1 provides a breakdown of response categories. Agreement was highest for the statement “Data sharing is good for science,” with 97% of participants agreeing. Other widely endorsed beliefs included “Data sharing will help increase my citations” (72% of researchers agreed) and “Data sharing is good for my career” (69% agreed).

### Research question 2: agreement with barriers to data sharing

**Fig. 2:**
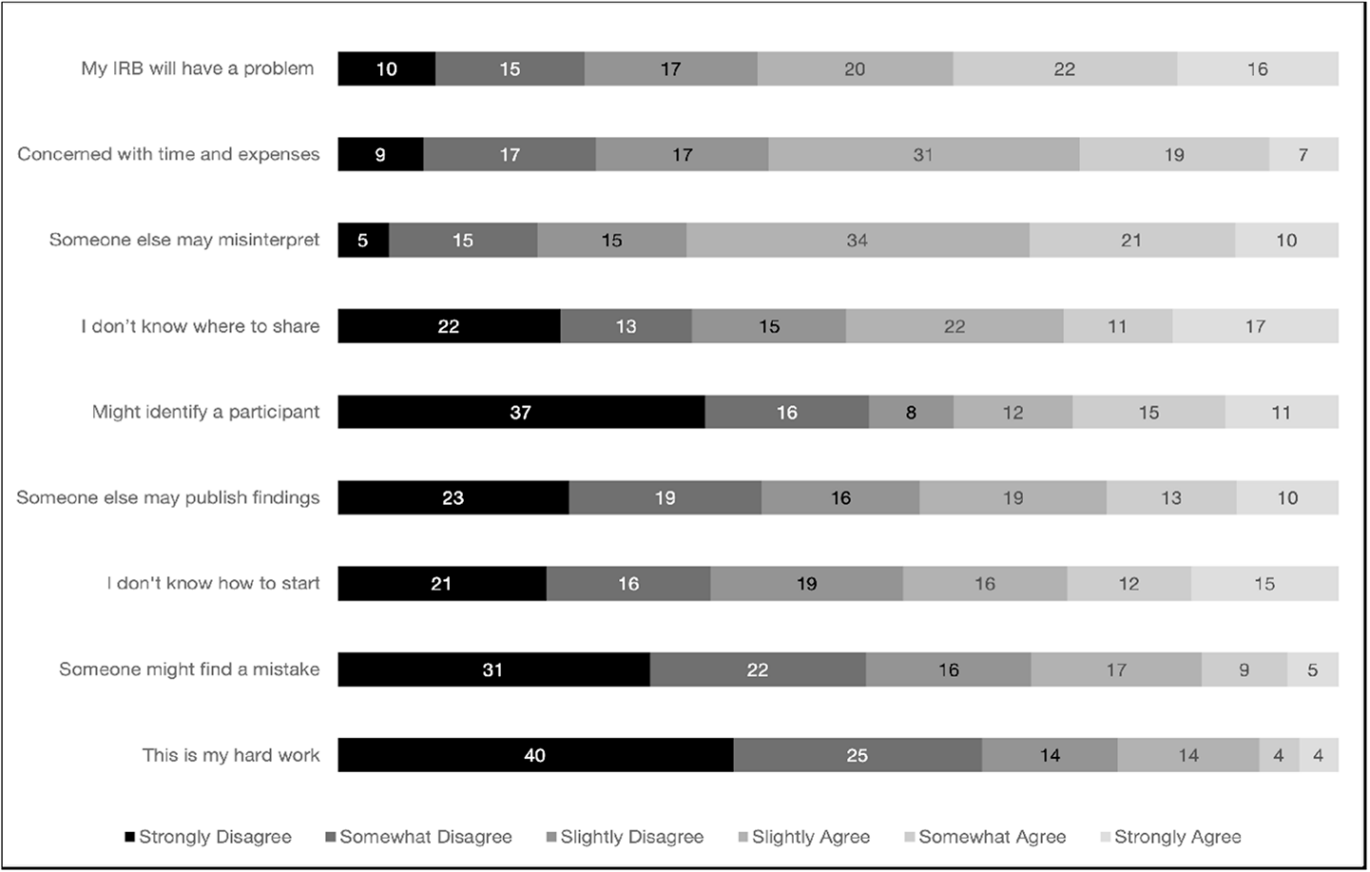
Percentage of the sample indicating each level of agreement with each data sharing barrier

Respondents’ agreement with statements reflecting barriers to data sharing is presented in Table 1; Fig. 2. Agreement with barrier statements varied (mean range = 2.30–3.81). Examining the breakdown of responses, the most frequently endorsed barriers included “My IRB will have a problem with me sharing data” (57%), “Data sharing is time-consuming and expensive” (57%), and “If someone reuses my data, they may misinterpret it” (65%). Other notable barriers included uncertainty about where to share data (49%) and concerns about participant confidentiality (38%). Less frequently endorsed barriers included fears of others publishing key findings first (42%), discovery of mistakes in the data (31%), and reluctance to share data, citing “this is my hard work” (22%).

### Research question 3: data sharing experience

*Future Data Sharing Plans.* Participants’ future data sharing plans were associated with their previous sharing experiences. As we described earlier, of the 177 participants analyzed, 72 (40.6%) reported sharing only with a collaborator, 74 (41.8%) reported sharing in a repository, and 31 (17.5%) reported never sharing. Among those who had only shared data with collaborators, 28% planned to share data from a current study. This proportion was 62% for those who had shared data in a repository, and only 11% for those who had never shared data. A chi-square test confirmed a significant relationship between past data sharing experiences and future intentions (χ²(2, 177) = 15.01, *p* =.005).

**Fig. 3:**
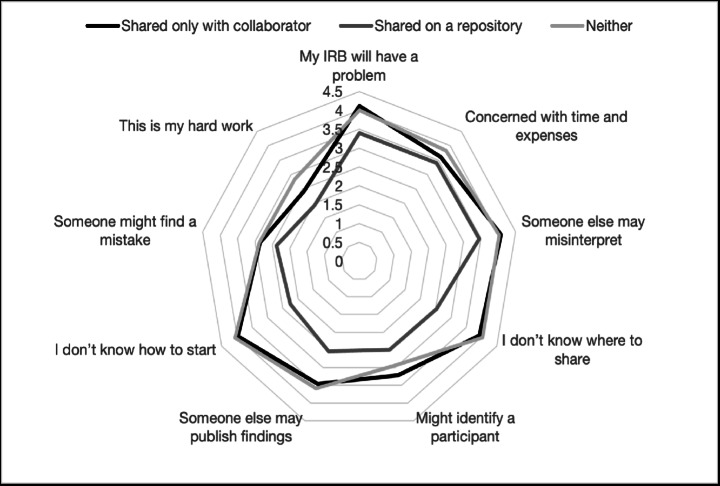
Mean barrier endorsement for each data sharing experience category (never shared, shared only with a collaborator, shared on a repository). An asterisk represents a barrier with a statistically significant between-group main effect (*p* <.05)

*Perceived Barriers*. The perceived barriers to data sharing varied significantly based on participants’ previous data sharing experiences. Table 3; Fig. 3 present the mean agreement levels for barriers across three groups: those who had only shared with collaborators, those who had shared in a repository, and those who had never shared data. To aid interpretation, we first provide the means of each item by group, followed by the results of the corresponding ANOVAs. Specifically, for each ANOVA, we provide the F-test of the main effect of group membership on the mean agreement with each item.

**Table 3:** Results of the ANOVAs comparing means for each data sharing question by group membership

Question	Shared with Collaborator M(SD)	Shared in a Repository M(SD)	Never Shared Data M(SD)	F	*p*	Effect Size Collaborator - Never shared	Effect Size Repository - Never shared
Good for my career	4.04 (1.34)	4.34 (1.33)	3.97 (1.28)	1.28	.280	0.05	-0.29
Increase citations	4.11 (1.55)	3.40 (1.49)	4.00 (1.69)	0.54	.585	-0.09	0.47
Good for science	5.12 (0.90)	5.58 (0.76)	5.19 (1.05)	5.42	.005*	-0.08	-0.42
IRB may have a problem	3.97 (1.55)	2.26 (1.32)	4.06 (1.69)	4.17	.017*	0.06	1.14
Time consuming/expensive	3.21 (1.84)	2.50 (1.76)	2.94 (1.91)	1.07	.345	-0.20	0.32
Someone may misinterpret	3.46 (1.62)	2.54 (1.52)	3.58 (1.73)	4.71	.010*	0.09	0.78
Unsure where to share	3.89 (1.24)	4.11 (1.35)	4.03 (1.27)	16.70	< .001*	0.08	-0.05
Re-identification concerns	3.93 (1.67)	2.53 (1.47)	4.03 (1.80)	2.79	.064	0.05	0.82
Someone might publish first	2.85 (1.65)	2.38 (1.44)	2.87 (1.36)	7.76	< .001*	0.01	0.30
I don’t know how to start	2.43 (1.41)	1.95 (1.28)	2.84 (1.55)	29.70	< .001*	0.24	0.52
Someone might find a mistake	3.60 (1.38)	3.40 (1.44)	3.83 (1.23)	2.10	.126	0.15	0.28
My hard work	4.08 (1.34)	3.45 (1.29)	4.03 (1.30)	5.08	.007*	-0.04	0.41

Note. Item names are presented in shorthand, and are in the same order as Table 1. F is the result of the ANOVA omnibus test for the main effect of group. Effect sizes Cohen’s *d*, calculated as the mean difference between groups divided by the overall standard deviation (reported in Table 1)

For example, for the barrier “My IRB may have a problem with me sharing data,” the mean for participants who had shared with collaborators was 3.97, for those who had shared in a repository was 2.26, and for those who had never shared data, it was 4.06. The ANOVA for this item was statistically significant (*F*(2, 172) = 4.17, *p* =.017), indicating a significant effect of data sharing experience on perceived barriers. The last two columns of Table 3 present effect size estimates, calculated as Cohen’s d. The difference between those who shared data with a collaborator and those who never shared was *d* = 0.06, while the difference between those who shared in a repository and those who never shared was *d* = 1.14.

Overall, six out of the nine barriers showed significant differences across the groups. For clarity, Fig. 3 visually plots the between-group differences. Among the barriers with significant differences, participants who had shared data in a repository consistently reported lower levels of agreement with the perceived barriers compared to those who had only shared with collaborators or those who had never shared data at all.

## Discussion

The purpose of this study was to examine education researchers’ beliefs, barriers, and past experiences with data sharing. Our results indicate that while most education researchers recognize the benefits of sharing data, significant barriers persist that discourage them from adopting data sharing practices in their own work.

A majority of respondents expressed positive beliefs about data sharing, with nearly all (97%) agreeing that “data sharing is good for science.” This is notably higher than similar findings in previous studies. For instance, Makel et al. (2021) found that 75% of participants agreed that data sharing was good for science, while Houtkoop et al. (2018) and Fleming et al. (2022) reported agreement rates of 77% and 72%, respectively. A key difference between the present study and these earlier studies is the level of familiarity with data sharing. In prior research, a large portion of the samples had little to no experience with data sharing—80% of Makel et al.‘s (2021) sample had never shared data, and 89% of Fleming et al.‘s (2022) sample reported the same. In contrast, 42% of our respondents had previous experience sharing data in a repository. This higher baseline familiarity may influence the interpretation of our findings and suggests a potentially more informed perspective on the practice of data sharing.

For research question two, which explored perceived barriers to data sharing, our results align with previous studies in some respects, but also reveal domain-specific challenges for education researchers. A common barrier across fields was the perception that “data sharing is time-consuming and expensive,” which 57% of our participants agreed with. This is similar to the 55% agreement rate reported by Houtkoop et al. (2018). However, the higher level of data sharing experience in our sample makes this barrier particularly noteworthy, as participants who have shared data should be more familiar with the associated costs and time commitments.

The most frequently endorsed barrier in our study was the fear that data might be misinterpreted, with 65% of researchers expressing at least some concern. This finding mirrors the results of Houtkoop et al. (2018), who found 49% of psychology researchers shared this fear. The higher prevalence of this concern in our sample may be attributed to the nature of education research, which often involves school-based studies and complex data, including ethical considerations related to child participants. Notably, 69% of researchers reported the inclusion of participants under age 18, which may heighten concerns about misinterpretations of data.

Another salient barrier in our sample was the concern that the Institutional Review Board (IRB) might object to data sharing, with 57% of participants agreeing. While this barrier has not been extensively studied in previous literature, it underscores the unique challenges faced by education researchers, particularly when it comes to ensuring participant privacy and adhering to ethical guidelines. Along these lines, concerns about re-identifying participants were endorsed by 38% of our respondents. This was much higher than the 4% reported by Houtkoop et al. (2018) on a similar item, that anonymity could not be guaranteed if the data were shared. This suggests that issues of confidentiality and anonymity are more pressing in education research, and speaks to the importance of examining barriers with domain specificity; barriers that were salient for this population of education researchers were less of an issue in other fields.

### Data sharing experience

For our third research question, we examined the relationship between data sharing experience and perceived barriers. Our results indicate that researchers who had shared data in a repository reported significantly lower levels of agreement with several barriers compared to those who had shared data only with collaborators or had never shared. This aligns with the theory of planned behavior (Ajzen 1985), which states that perceived behavioral control—confidence in one’s ability to perform a behavior—affects both intentions and actions. Posited to be at play in data sharing behaviors by Fleming et al. (2022), the theory of planned behavior in this context suggests that researchers who have shared data in a repository likely perceive greater control over the process, as they have already navigated the initial challenges, such as knowing where to share data and how to get started. These findings suggest that one important avenue for promoting data sharing is to support researchers in overcoming the initial barriers to getting started. A recent guide from the Institute of Education Sciences (Neild et al. 2022) offers a valuable resource for researchers seeking guidance on how to begin sharing their data, and similar resources could play a crucial role in helping others take the first step. We also provide some additional resources that are discussed in the next section.

In addition to barriers, we also examined researchers’ intent to share in the future, and found that experience with data sharing plays a significant role in shaping future intentions. Our findings suggest that experience perhaps plays a smaller role than we might have expected: only 62% of repository sharers report planning to share data from their current study in the future. However, this is substantially higher than those who have only shared data with collaborators (28%), and higher than those who have never shared (11%). This indicates that, while experience is an important driver of intent to participate in the practice in the future, it is far from the only factor related to researchers’ engagement in data sharing. However, it is worth noting that researchers who share data on repositories only do so for some projects and not necessarily for all. The way this question is asked may present a limitation to our understanding of future intent to share. For example, experienced data sharers may have said no to sharing data from a current study simply because of the current study they are working on. That is not to say that they will never share data again.

### Addressing barriers and providing resources

This study explored various potential barriers to data sharing among education researchers. Notably, each barrier identified was endorsed by at least some respondents. Even the least frequently endorsed barrier, “this is my data,” was agreed upon by 22% of the sample. Although these findings do not establish causality, they provide valuable insights into the challenges education researchers face when it comes to data sharing. In this section, we will briefly highlight existing resources designed to address three of the most prominent barriers identified in our study. For additional support regarding other barriers that we do not directly address, here, we recommend work by Van Dijk et al. (2021); Logan et al. (2021); Shero and Hart (2020a, b).

#### If I share my data, it might be possible to identify a participant

Researchers have developed many techniques for data de-identification that can preserve the key facets of the data while minimizing the likelihood of re-identification. For example, with continuous data (e.g., salary), researchers can use a recoding process to round data to the nearest $10,000, reducing the likelihood that an individual can be identified by exact salary dollar amount (Angiuli et al. 2015). Another class of methods are perturbative methods. These methods involve techniques such as data swapping or adding random error to the data points (e.g., Domingo-Ferrer and Torra 2003). For an introduction to basic de-identification techniques and how to apply them, see Shero et al. (2024). To read more about how to determine the re-identification risk for a given dataset, see the recent paper by Morehouse et al. (2024). In addition, many data repositories will allow data to be shared under a restricted use license, such that it can only be accessed after a request has been submitted and reviewed by the original research team (Logan et al. 2021). In many cases, researchers can meet data sharing expectations by sharing the means, standard deviations, and correlation matrices for all key variables. Many analyses can be conducted with a correlation matrix as input, and research teams can also use these summary statistics to create synthetic data (Jordon et al., n.d.).

#### I don’t know where to share my data

Data repositories can generally be categorized into two types: domain-general and domain-specific. Domain-general repositories accept data across multiple disciplines and subjects (e.g., Figshare, OSF), whereas domain-specific repositories house data from one particular research field (e.g., LDbase, which houses quantitative data related to learning and development), data type, (e.g., The Qualitative Data Repository, which holds data generated from qualitative or mixed methods studies), or both (e.g., Databrary, which houses video and audio files related to children’s development). The National Institutes of Health maintains a list of recommended repositories, depending on the type of funding and subject area. Starting with these resources is often the best approach, as they point to well-established and maintained repositories that are widely recognized in the research community. Researchers may also find it helpful to consult with a librarian for guidance in selecting a repository best suited to their needs.

#### If someone reuses my data, they May misinterpret it

The concern about misinterpretation of data is valid, but it can be mitigated through careful documentation and transparency. Published research typically adheres to rigorous standards, ensuring that the data is of sufficient quality to support accurate interpretation. Researchers can further reduce the risk of misinterpretation by providing clear and detailed descriptions of the sample, study protocols, measures, and other methodological aspects. By doing so, data re-users are equipped with a comprehensive understanding of how the data was collected, analyzed, and should be used. One of the principles of data sharing is that data should be Reusable, and one part of that principle is that Data should always be shared with study documentation that facilitates interpretation (*Common Data Elements*, 2021). Providing adequate metadata such as codebooks, summary documents, study protocols, analysis plans, and records documenting missingness further reduces the risk of misinterpretation (Lewis 2024; Logan et al. 2021; Neild et al. 2022).

### Limitations and future research

Several limitations are present in our study. First, we sought to examine barriers to data sharing, but we did so by asking researchers to agree with general statements about their beliefs. Although this method is in alignment with other literature in the field (Houtkoop et al. 2018; Makel et al. 2021), this work does not directly test whether barriers are functioning to keep researchers from sharing their data. Future researchers could examine barriers more directly by directly asking participants whether and how these barriers stop researchers from engaging in data sharing. Additionally, this information would inform how we should train the future generation of special education researchers. Houtkoop et al. (2018), who reported that 63% of researchers in psychology said they would be more likely to share if someone would show them how. This suggests that direct instruction in data sharing could be an effective method at improving researchers’ attitudes and removing barriers to data sharing, which future research could examine.

Next, the present study uses a researcher-developed survey that has not been psychometrically evaluated. Though it is common in meta-scientific work to create new items and measures, any time research uses measures that have not been well validated it adds some uncertainty to any research findings (Flake and Fried 2020). In this study, we did see findings that align with other similar questions in different fields, minimizing these validity concerns. However, this concern generally opens several areas for future research. General psychometric analysis could be conducted, or a validity study comparing self-rating on items like those included in this study and other observational measures of data sharing behaviors. Further, items about barriers to engagement or use of metascience practices could be explored to determine whether items could be combined to create higher-order composites that more accurately capture participant beliefs and behaviors.

Another notable limitation of the present study relates to external validity. This project relates the findings of a relatively small sample of participants (*n* = 178), and resulted from recruitment via 1,114 direct emails (250 / 1,114 = 23% return rate, though this is an overestimate due to the listservs and social media recruitment methods). This sample is biased in a few important ways. Given that they responded to our survey, we can assume that they may be more accepting of open science practices. Next, as noted in the methods section, one recruitment strategy was to write to researchers who had received federal funds. Therefore, compared to the general population of education researchers, this group of participants more likely to be federally funded. Next, participants were recruited via survey invitations sent to researchers involved in the Special Education Research Accelerator (SERA), and therefore our results include an over-representation of researchers from the field of special education. Finally, because SERA is a platform specifically for conducting open science studies, our sample is biased in favor of those researchers that are aware of, and likely support, open science practices. This is evidenced by the fact that 42% of our sample reported having previously shared data in a repository, which is far higher than what has been reported in other samples. It will be important for future work to collect data from a more normative range of researchers in the field.

Finally, the present study is correlational. Correlational studies determine association between variables but cannot make causal claims. Therefore, we cannot rule out possible confounding variables influencing the relations between researchers’ attitudes and experiences with their data sharing practices; it is possible that researchers who already had fewer limiting beliefs were more likely to participate in the practice of data sharing. Currently, the field is in a state of change, with the implementation of new open data mandates (e.g., NIH, IES, Gates Foundation). These historical changes warrant the continued study of researchers’ attitudes and behaviors with regards to participation in data sharing and suggest there may be important future longitudinal or causal work that could be done to determine best practices for improving researchers’ uptake of these practices.

## Electronic supplementary material

Below is the link to the electronic supplementary material.

Supplementary Material 1

Supplementary Material 3

## Data Availability

There data and code used in the present study are available on LDbase at https://ldbase.org/projects/dbf4158d-5e7b-407e-993d-da559c189a93
