# Supplementary material for: Education researchers’ beliefs and barriers towards data sharing
Source: Qual Quant. 2025 Apr 29;59(5):4061–75. doi: 10.1007/s11135-025-02188-6 (PMC12476402; doi:10.1007/s11135-025-02188-6)
Supplement: Supplementary file 1 — Supplementary Material 1 [file 11135_2025_2188_MOESM1_ESM.docx]

Appendix

Data Sharing Beliefs and Barriers

This research question was examined in an academic poster, but we realized with the help of anonymous reviewers that this was not very methodologically sound nor well motivated in the literature. We include it for transparency.

**Barrier Differences by Sharing Attitude**

Research Question: Is there a difference between the perceived barriers for researchers who identify with positive statements compared to those who do not?

This research question assessed if there was a difference in perceived barriers between researchers who identify with positive data sharing beliefs and those who do not. To do so, two comparison groups were created, the believers and skeptics. Of the 178 participants, 128 agreed with all three positive statements about data sharing, which we labeled the believers. The remaining 50 participants disagreed with at least one positive statement, which we labeled the skeptics.

Descriptive analyses were run to compare groups. On average, believers (*M* = 8.31, *SD* = 7.24) had about one more year of experience than skeptics (*M* = 9.55, *SD* = 6.69). Of researchers who reported education as their field, 70% were believers and 30% were skeptics. Academia was the most common workplace reported, where 73% of researchers were believers and 27% were skeptics. When asked if the researcher had plans to share data at the completion of a current project, 86% of believers and 14% of skeptics said yes. Believers tended to on average agree more with positive sharing statements compared to skeptics, except for item 2 “data sharing will increase my citations “(believers *M* = 3.51; skeptics *M* = 4.5). Skeptics tended to on average agree more with barrier statements compared to believers, except for item 7 “I don’t know where to share my data” (believers *M* = 4.31; skeptics *M* = 3.22) and item 12 “these data exist because of my hard work” where they were about equal (believers *M* = 3.81; skeptics *M* = 3.82).

Each item representing a perceived barrier was assessed individually to compare the mean difference between believers and skeptics. A series of ANOVAs revealed significant mean differences for multiple of the perceived barriers. Figure A.1 visualizes the differences between the groups for each barrier. The following items resulted in a significant difference between the believers and skeptics groups: “My IRB will have a problem” (*F* (1, 169) = 13.42, *p* = < .001), “If I share my data, it might be possible to identify a participant” (*F* (1, 169 ) = 7.79, *p* = < .001 ), “If I share my data, someone might publish my key findings before I do” (*F* (1, 169 ) = 8.78, *p* = < .001 ), “I don’t want to share my data because someone might find a mistake” (*F* (1, 169) = 4.37, *p* =< .01 ), and “These data exist because of my hard work, why would I share so someone else benefits?” (*F* (1, 169) = 8.12 , *p* = < .001). In each of these items’ participants deemed as believers in data sharing identified significantly less than skeptics of data sharing with the barrier presented. The discussion section of this paper provides resources for each of these salient barriers.”

**Figure A.1**

Mean barrier endorsement for each data sharing belief category (skeptic or believer)*.* An asterisk represents a survey item with a between-group statistically significant mean difference (*p* < .05)*.*
