# Supplementary material for: Education researchers’ beliefs and barriers towards data sharing
Source: Qual Quant. 2025 Apr 29;59(5):4061–75. doi: 10.1007/s11135-025-02188-6 (PMC12476402; doi:10.1007/s11135-025-02188-6)
Supplement: Supplementary file 2 — Supplementary Material 3 [file 11135_2025_2188_MOESM2_ESM.docx]

R code for analyses used for “Education Researchers’ Beliefs and Barriers towards Data Sharing”

#install packages

install.packages("tidyverse")

library(tidyverse)

library(ggplot2)

library(dplyr)

#upload data d

s<- read.csv("data_sharing_data.csv")

#%%%%%%%%%%%%%%%%%%%%%%%%%%%%%%%%%%%%%%%%%%%%%%%%%%%%%%%%%%

RQ1 and 2 agreement with positive statements ane barriers to data sharing

#%%%%%%%%%%%%%%%%%%%%%%%%%%%%%%%%%%%%%%%%%%%%%%%%%%%%%%%%%%

mean(ds$Q5d_1, na.rm=TRUE) sd(ds$Q5d_1, na.rm=TRUE)

mean(ds$Q5d_2, na.rm=TRUE) sd(ds$Q5d_2, na.rm=TRUE)

mean(ds$Q5d_3, na.rm=TRUE) sd(ds$Q5d_3, na.rm=TRUE)

mean(ds$Q5d_4, na.rm=TRUE) sd(ds$Q5d_4, na.rm=TRUE)

mean(ds$Q5d_5, na.rm=TRUE) sd(ds$Q5d_5, na.rm=TRUE)

mean(ds$Q5d_6, na.rm=TRUE) sd(ds$Q5d_6, na.rm=TRUE)

mean(ds$Q5d_7, na.rm=TRUE) sd(ds$Q5d_7, na.rm=TRUE)

mean(ds$Q5d_8, na.rm=TRUE) sd(ds$Q5d_8, na.rm=TRUE)

mean(ds$Q5d_9, na.rm=TRUE) sd(ds$Q5d_9, na.rm=TRUE)

mean(ds$Q5d_10, na.rm=TRUE) sd(ds$Q5d_10, na.rm=TRUE)

mean(ds$Q5d_11, na.rm=TRUE) sd(ds$Q5d_11, na.rm=TRUE)

mean(ds$Q5d_12, na.rm=TRUE) sd(ds$Q5d_12, na.rm=TRUE)

#%%%%%%%%%%%%%%%%%%%%%%%%%%%%%%%%%%%%%%%%%%%%%%%%%%%%%%%%%%

#RQ3 differences on items between share type

#%%%%%%%%%%%%%%%%%%%%%%%%%%%%%%%%%%%%%%%%%%%%%%%%%%%%%%%%%%

#create new variable for type of type of sharing experience

#1 = collab only, 2 = repository, 3 = neither

ds <- ds%>%

mutate(share_type = case_when( Q5a>=2 & Q5b==1 ~ 1, Q5b>=2 ~ 2, Q5a==1 & Q5b==1 ~ 3 ))

table(ds$ds.share_type)

one.way.rq3.2 <- aov(ds.Q5d_2 ~ ds.share_type, data = ds) summary(one.way.rq3.2 ) coefficients(one.way.rq3.2 )

#beliefs

ds$ds.share_type <- as.factor(ds$ds.share_type) table(ds$ds.share_type)

#Q5d1

good for science

one.way.A <- aov(ds.Q5d_1 ~ ds.share_type, data = ds) summary(one.way.A) TukeyHSD(one.way.A)

#Q5d3

#good for career

one.way.B <- aov(ds.Q5d_3 ~ ds.share_type, data = ds) summary(one.way.B) TukeyHSD(one.way.B)

Q5d7

#increase citations

one.way.C <- aov(ds.Q5d_7 ~ ds.share_type, data = ds) summary(one.way.C) TukeyHSD(one.way.C)

#barriers

#Q5d2 My IRB will have a problem with me sharing data.

one.way.2 <- aov(ds.Q5d_2 ~ ds.share_type, data = ds)
summary(one.way.2)
TukeyHSD(one.way.2)

#Q5d4

#I don’t know how to start sharing my data.

one.way.4 <- aov(ds.Q5d_4 ~ ds.share_type, data = ds)

summary(one.way.4)
coefficients(one.way.4)

#Q5d5

#If I share my data, it might be possible to identify a participant.

one.way.5 <- aov(ds.Q5d_5 ~ ds.share_type, data = ds)

summary(one.way.5)
coefficients(one.way.5)

#Q5d6

#If I share my data, someone might publish my key findings before I do.

one.way.6 <- aov(ds.Q5d_6 ~ ds.share_type, data = ds)

summary(one.way.6)
coefficients(one.way.6)

#Q5d8

#I don’t know where to share my data.

one.way.8 <- aov(ds.Q5d_8 ~ ds.share_type, data = ds)

summary(one.way.8)
coefficients(one.way.8)

#Q5d9

#I don’t want to share my data because someone might find a mistake.

one.way.9 <- aov(ds.Q5d_9 ~ ds.share_type, data = ds)

summary(one.way.9)
coefficients(one.way.9)

#Q5d10

#These data exist because of my hard work, why would I share so someone else benefits?

one.way.10 <- aov(ds.Q5d_10 ~ ds.share_type, data = ds)

summary(one.way.10)
coefficients(one.way.10)

#Q5d11

#Data sharing is time consuming and expensive.

one.way.11 <- aov(ds.Q5d_11 ~ ds.share_type, data = ds)

summary(one.way.11)
coefficients(one.way.11)

#Q5d12

#If someone reuses my data, they may misinterpret

one.way.12 <- aov(ds.Q5d_12 ~ ds.share_type, data = ds)

summary(one.way.12)
coefficients(one.way.12)


#demographic information of entire sample
## field
table(ds$Q1a)
## area of research (Q1b)
sum(ds$Q1b_1,na.rm=TRUE)
sum(ds$Q1b_2,na.rm=TRUE)
sum(ds$Q1b_3,na.rm=TRUE)
sum(ds$Q1b_4,na.rm=TRUE)
sum(ds$Q1b_5,na.rm=TRUE)
sum(ds$Q1b_6,na.rm=TRUE)
sum(ds$Q1b_7,na.rm=TRUE)
## workplace
table(ds$Q1c)
## funded IES
table(ds$Q1e_1)
## funded NIH
table(ds$Q1e_2)
## funded NSF
table(ds$Q1e_3)
## add all three funding sites and get sums
Q1e_sum <- ds$Q1e_1+ds$Q1e_2+ds$Q1e_3
table(Q1e_sum)
## under 18
table(ds$Q1f)
## types of data (Q1g)
sum(ds$Q1g_1,na.rm=TRUE)
sum(ds$Q1g_2,na.rm=TRUE)
sum(ds$Q1g_3,na.rm=TRUE)
sum(ds$Q1g_4,na.rm=TRUE)
sum(ds$Q1g_5,na.rm=TRUE)
sum(ds$Q1g_6,na.rm=TRUE)
sum(ds$Q1g_7,na.rm=TRUE)
sum(ds$Q1g_8,na.rm=TRUE)
##reuse your own data?
table(ds$Q1h)
##used data from a repository
table(ds$Q1i)
## plan to share
table(ds$Q5c)
## years of experience
mean(ds$Q1d, na.rm=TRUE)
sd(ds$Q1d, na.rm=TRUE)
## mean and SD for items
sapply(ds, mean)
summary(ds)
sd(ds$Q1d, na.rm=TRUE)

# frequency by Q5a Q5b
table(ds$Q5a)
table(ds$Q5b)


## demographics by share_type var
##field
table(ds$`share_type`, ds$Q1a)
## area of research (Q1b)
table(ds$`share_type`, ds$Q1b_1)
table(ds$`share_type`, ds$Q1b_2)
table(ds$`share_type`, ds$Q1b_3)
table(ds$`share_type`, ds$Q1b_4)
table(ds$`share_type`, ds$Q1b_5)
table(ds$`share_type`, ds$Q1b_6)
table(ds$`share_type`, ds$Q1b_7)
## workplace
table(ds$`share_type`, ds$Q1c)
## funded IES
table(ds$`share_type`, ds$Q1e_1)
## funded NIH
table(ds$`share_type`, ds$Q1e_2)
## funded NSF
table(ds$`share_type`, ds$Q1e_3)
## all three grant sites
ds3 <- cbind(ds, Q1e_sum)
table(ds3$share_type, ds3$Q1e_sum)
## under 18
table(ds$`share_type`, ds$Q1f)
## types of data (Q1g)
table(ds$`share_type`, ds$Q1g_1)
table(ds$`share_type`, ds$Q1g_2)
table(ds$`share_type`, ds$Q1g_3)
table(ds$`share_type`, ds$Q1g_4)
table(ds$`share_type`, ds$Q1g_5)
table(ds$`share_type`, ds$Q1g_6)
table(ds$`share_type`, ds$Q1g_7)
table(ds$`share_type`, ds$Q1g_8)
##reuse your own data?
table(ds$`share_type`, ds$Q1h)
##used data from a repository
table(ds$`share_type`, ds$Q1i)
## shared with collaborator
table(ds$'share_type', ds$Q5a)
## shared on a repository
table(ds$'share_type', ds$Q5b)
## plan to share
table(ds$`share_type`, ds$Q5c)
## years of experience
ds %>%
 group_by(agreementfinal) %>%
 summarise_at(vars(Q1d), list(name = mean), na.rm=TRUE)
ds %>%
 group_by(agreementfinal) %>%
 summarise_at(vars(Q1d), list(name = sd), na.rm=TRUE)
ds %>%
 group_by(agreementfinal) %>%
 summarise_at(vars(Q1d), list(name = median), na.rm=TRUE)

table(ds$share_type)
data_try <- data.frame(ds$ID, ds$Q1d, ds$agreementfinal, ds$share_type)


## mean agreement grouped by share_type
ds %>%
 group_by(share_type) %>%
 summarise_at(vars(Q5d_1), list(name = mean), na.rm=TRUE)
ds %>%
 group_by(share_type) %>%
 summarise_at(vars(Q5d_2), list(name = mean), na.rm=TRUE)
ds %>%
 group_by(share_type) %>%
 summarise_at(vars(Q5d_3), list(name = mean), na.rm=TRUE)
ds %>%
 group_by(share_type) %>%
 summarise_at(vars(Q5d_4), list(name = mean), na.rm=TRUE)
ds %>%
 group_by(share_type) %>%
 summarise_at(vars(Q5d_5), list(name = mean), na.rm=TRUE)
ds %>%
 group_by(share_type) %>%
 summarise_at(vars(Q5d_6), list(name = mean), na.rm=TRUE)
ds %>%
 group_by(share_type) %>%
 summarise_at(vars(Q5d_7), list(name = mean), na.rm=TRUE)
ds %>%
 group_by(share_type) %>%
 summarise_at(vars(Q5d_8), list(name = mean), na.rm=TRUE)
ds %>%
 group_by(share_type) %>%
 summarise_at(vars(Q5d_9), list(name = mean), na.rm=TRUE)
ds %>%
 group_by(share_type) %>%
 summarise_at(vars(Q5d_10), list(name = mean), na.rm=TRUE)
ds %>%
 group_by(share_type) %>%
 summarise_at(vars(Q5d_11), list(name = mean), na.rm=TRUE)
ds %>%
 group_by(share_type) %>%
 summarise_at(vars(Q5d_12), list(name = mean), na.rm=TRUE)
